# Toward a broader view of mechanisms of drug cardiotoxicity

**DOI:** 10.1016/j.xcrm.2021.100216

**Published:** 2021-03-16

**Authors:** Polina Mamoshina, Blanca Rodriguez, Alfonso Bueno-Orovio

**Affiliations:** 1 Deep Longevity Limited, Hong Kong; 2 Department of Computer Science, BHF Centre of Research Excellence, University of Oxford, Oxford, UK

**Keywords:** cardiotoxicity, side effects, adverse reactions, cell signaling, mechanisms of toxicity

## Abstract

Cardiotoxicity, defined as toxicity that affects the heart, is one of the most common adverse drug effects. Numerous drugs have been shown to have the potential to induce lethal arrhythmias by affecting cardiac electrophysiology, which is the focus of current preclinical testing. However, a substantial number of drugs can also affect cardiac function beyond electrophysiology. Within this broader sense of cardiotoxicity, this review discusses the key drug-protein interactions known to be involved in cardiotoxic drug response. We cover adverse effects of anticancer, central nervous system, genitourinary system, gastrointestinal, antihistaminic, anti-inflammatory, and anti-infective agents, illustrating that many share mechanisms of cardiotoxicity, including contractility, mitochondrial function, and cellular signaling.

## Graphical abstract

**Figure:**
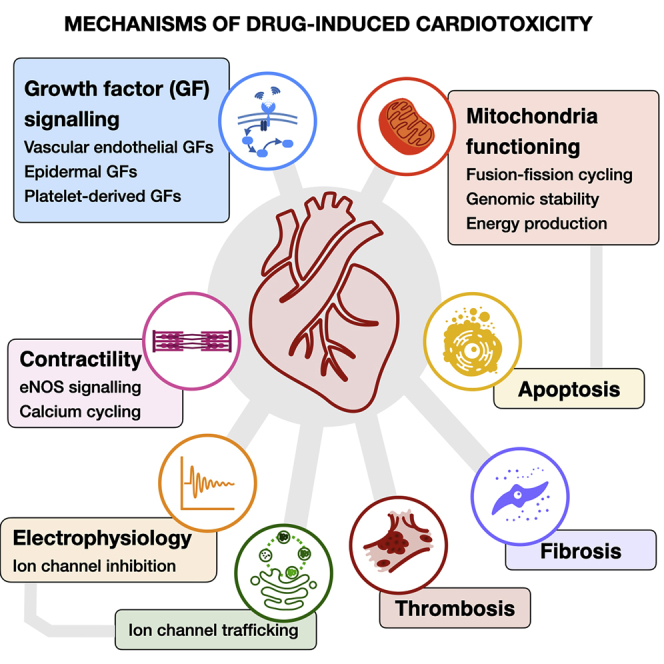

## Teaser

Adverse cardiac effects remain a top reason for drug withdrawal from the market. Mamoshina et al. provide a comprehensive review of drug-protein interactions involved in cardiotoxic drug response, covering anticancer, central nervous system, genitourinary system, gastrointestinal, antihistaminic, anti-inflammatory, and anti-infective agents and identifying avenues into drug discovery, safety pharmacology, and pharmacovigilance studies.

## Introduction

**Figure 1:**
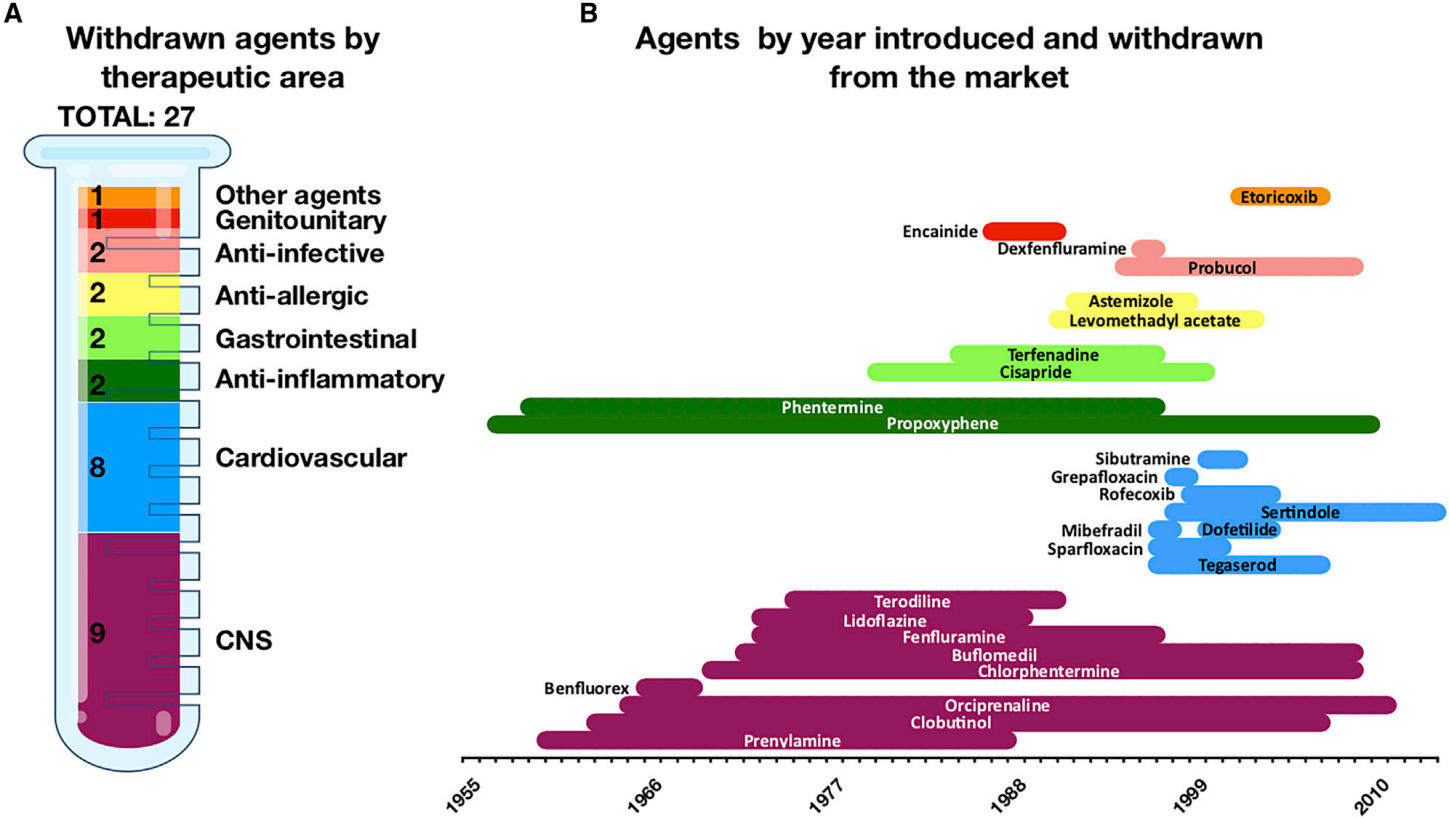
Drugs withdrawn from market due to cardiotoxicity up to May 2020 (A) Number of drugs withdrawn according to therapeutic area. (B) Lifespan of withdrawn agents. CNS, central nervous system.

Concerns regarding cardiac safety are among the top reasons for drug withdrawal from clinical trials and the market. Strict examination of cardiac safety liabilities has resulted in a sustained increase in attrition rates at all phases of drug development over the last decades.^1^ This increased attrition rate is illustrated in Figure 1, summarizing by therapeutic area all drugs withdrawn from the market due to cardiac safety concerns, as well as their commercialization lifespans. The latter have been greatly reduced over the last decades. Although this process of continuous pharmacovigilance greatly reassures the safety of new commercialized compounds, it also highlights that many constituent molecular processes underlying cardiotoxicity have yet to be comprehensively understood. This is substantially challenging for several scientific reasons. First, the spectrum of cardiotoxicity is broad, spanning from arrhythmia, to myocardial dysfunction, to terminal heart failure. In addition, the extent of the effects depends on exposure and varies among patients. Finally, adverse drug effects have been shown to depend on the gender, age, and genetic background of the individual.^2^, ^3^, ^4^

Drug-induced cardiotoxicity thus imposes substantial limits on drug development, as well as in the clinical management of existing drugs.^1^ Particularly critical across safety liabilities is the risk to induce potentially lethal arrhythmias via direct interactions with cardiac electrophysiology. However, recent studies have shown that a considerable number of drugs in use can also disrupt cardiac function by impairing myocardial metabolism and cardiac structure. These include anticancer therapies associated with myocardial apoptosis,^5^ neurodegenerative disease agents with severe risk of fibrotic valvular heart disease,^6^ or antibacterial^7^ and antiviral^8^ treatments leading to mitochondrial damage.

Multiple drug classes have the potential to induce cardiovascular adverse effects in patients. In this review, we summarize the drug-protein mechanisms by which drug-induced cardiotoxicity may develop. We provide a detailed review of 78 drugs with known cardiac adverse effects, 27 of which have been withdrawn from the market due to cardiotoxicity (Figure 1; Table 1). We cover adverse effects and cardiotoxic mechanisms of anticancer, central nervous system (CNS), genitourinary system, gastrointestinal, antihistaminic, anti-inflammatory, and anti-infective agents. We illustrate that they have common modes of action on cardiac function and on adverse cardiac events. Even though many of these points could be discussed in detail, we expect these findings will provide reliable guidance in identifying new critical pharmacophores, as well as key assays for the evaluation of drug-induced cardiotoxicity.

**Table 1:** List of compounds with their targets and cardiac side effects

Drug	Introduced–withdrawn	Mechanism of action with targets	Side effects on cardiac function	Mechanism of cardiac toxicity	References
**Antineoplastic agents**					

5-fluorouracil	2000–NA	DNA cross-linking	arrhythmias, myocardial ischemia, heart failure	TXA_2_ activation leading to cardiac remodeling, electrolyte imbalances	^9^^,^^10^
Arsenic trioxide	2000–NA	inhibition of TXNRD1; activation of IKBKB, JUN, MAPK3, MAPK1	QT prolongation, tachycardia	hERG trafficking inhibition	^11^^,^^12^
Bevacizumab	2016–NA	blocker of VEGFA	heart failure	disruption of cardiomyocyte survival via VEGF signaling inhibition	^13^
Bortezomib	2003–NA	inhibition of PSMB5, PSMB1	heart failure, arrhythmia	hERG trafficking inhibition	^14^^,^^15^
Cisplatin	1978–NA	DNA cross-linking	arrhythmias, myocardial ischemia, heart failure	TXA_2_ activation leading to cardiac remodeling, electrolyte imbalance	^9^^,^^16^
Cytarabine	1966–NA	DNA intercalation, inhibition of DNA polymerase β	bradycardia, heart failure, ischemia	unknown	^17^^,^^18^
Daunorubicin	1980–NA	inhibition of TOP2B	arrhythmias, heart failure	mitochondrial toxicity, oxidative stress leading to apoptosis	^19^^,^^20^
Dasatinib	2016–NA	Bcr-Abl kinase, EPHA2, LCK, YES1	heart failure	disruption of cardiomyocyte survival via VEGF signaling inhibition	^21^
Docetaxel	1991–NA	inhibition of β subunit of tubulin, Bcl-2	bradycardia, myocardial ischemia, heart failure	apoptosis of cardiac endothelial cells	^22^^,^^23^
Doxorubicin	1978–NA	DNA intercalation, inhibition of TOP2B	arrhythmias, heart failure	mitochondrial toxicity, oxidative stress leading to apoptosis	^19^^,^^24^
Idarubicin	1989–NA	inhibition of TOB2B	arrhythmias, heart failure	mitochondrial toxicity, oxidative stress leading to apoptosis	^19^
Imatinib	2016–NA	Bcr-Abl kinase, KIT, RET	systolic heart failure, heart failure, left ventricular dysfunction	release of Bcl-2 proteins leading to mitochondrial toxicity, oxidative stress leading to apoptosis, disruption of cardiomyocyte survival via VEGF signaling inhibition	^21^
Ipilimumab	2011–NA	inhibition of CTLA-4	lethal myocarditis	unknown	^25^^,^^26^
Lapatinib	2004–NA	blocking of EGFR, HER2	left ventricular ejection fraction, congestive heart failure	disruption of cardiomyocyte survival via EGF signaling inhibition	^27^
Nilotinib	2005–NA	inhibition of ABL1, blocking of KIT	myocardial ischemia	disruption of cardiomyocyte survival via VEGF signaling inhibition	^21^
Nivolumab	2017–NA	inhibition of PD-1	lethal myocarditis	unknown	^25^^,^^26^
Paclitaxel	1995–NA	inhibition of β subunit of tubulin, Bcl-2	bradycardia, myocardial ischemia, heart failure	apoptosis of cardiac endothelial cells	^28^^,^^29^
Romidepsin	2009–NA	inhibition of HDAC1, HDAC2, HDAC4, HDAC6, ABCC1	QT prolongation, myocardial infarction	hERG trafficking inhibition	^30^
Sorafenib	2006–NA	inhibition of BRAF kinase, FLT1, FLT3, FGFR1, KIT, PDGFRB, RAF1, RET, VEGFR2, VEGFR3	heart failure, myocardial ischemia, QT prolongation	disruption of cardiomyocyte survival via VEGF signaling inhibition	^31^
Sunitinib	2006–NA	inhibition of PDGFRB, FLT1, FLT3, FLT4, KDR, KIT, CSF1R, PDGFRA	long QT, left ejection fraction, myocardial infarction	disruption of cardiomyocyte survival via VEGF signaling inhibition, disruption of stress response via PDGF signaling inhibition, disruption of energy homeostasis and mitochondrial fusion-fission system via inhibition of AMPK signaling	^32^
Trastuzumab	2016–NA	blocker of HER2	heart failure, tachycardia	disruption of cardiomyocyte survival via EGF signaling inhibition	^33^^,^^34^
Vandetanib	2011–NA	inhibition of VEGFA, EGFR, PTK6, TEK	long QT	disruption of cardiomyocyte survival via VEGF and EGF signaling inhibition	^27^^,^^35^
Vinblastine	1960s–NA	inhibition of α, β, and δ subunits of tubulin	myocardial ischemia, heart failure	apoptosis of cardiac endothelial cells	^36^

**Anti-inflammatory agents**					

Diclofenac	1986–NA	inhibition of COX-1, COX-2, SCN4A, ASIC1; potentiation of ALOX5	myocardial infarction	blocking prostacyclin synthase	^37^^,^^38^
Etoricoxib	2002–2007	inhibition of COX-2	thrombotic events	blocking prostacyclin synthase	^37^^,^^38^
Ibuprofen	1978–NA	inhibition of COX-1, COX-2	myocardial infarction, hypertension	blocking prostacyclin synthase	^37^^,^^38^
Indomethacin	1966–NA	inhibition of COX-1, COX-2, PLA2G2A, GLO1; activation of PPARG	myocardial infarction	blocking prostacyclin synthase	^37^^,^^38^
Naproxen	1978–NA	inhibition of COX-1, COX-2	myocardial infarction	blocking prostacyclin synthase	^37^
Rofecoxib	1999–2004	inhibition of COX-2	myocardial infarction	blocking prostacyclin synthase	^39^

**Central nervous system agents**					

Benfluorex	1972–2009	blocking of 5-HT_2B_	valvular heart disease	HTR2B-induced activation of TGF-β signaling	^40^
Bupivacaine	1965–NA	inhibition of SCN10A	ventricular arrhythmias, myocardial depression	inhibition of voltage-gated sodium channel, mitochondrial toxicity	^41^
Chlorphentermine	1966–1969	blocking of 5-HTs	pulmonary heart disease	HTR2B-induced activation of TGF-β signaling	^42^
Clozapine	1991–NA	blocking of DRD2, HTR2A, DRD1, DRD3, DRD4, HTR1A, HTR1B, HTR1D, HTR1E, HTR2C, HTR3A, HTR6, HTR7, HRH1, HRH4, ADRA1A, ADRA1B, ADRA2A, ADRA2B, ADRA2C, CHRM1, CHRM2, CHRM3, CHRM4, CHRM5	myocarditis, cardiomyopathy	unknown	^43^^,^^44^
Cocaine	1884–NA	inhibition of SLC6A3, SLC6A2, SLC6A4, SCN5A	left ventricular hypertrophy, arrhythmias	inhibition of voltage-gated sodium channel, mitochondrial toxicity	^45^
Dexfenfluramine	1996–1997	inhibition of SLC6A4	valvular heart disease	HTR2B-induced activation of TGF-β signaling	^46^^,^^47^
Ergotamine	1925–NA	activation of ADRA1A, DRD2, HTR1B, HTR1D, HTR2A	valvular heart disease	induction of fibrosis via HTR2B-induced activation of TGF-β signaling	^48^
Fenfluramine	1973–1997	inhibition of SLC6A4, blocking of HTR2B	valvular heart disease	HTR2B-induced activation of TGF-β signaling	^47^^,^^49^
Fluoxetine	1990–NA	inhibition of SLC6A4	bradycardia	inhibition of I_CaL_, I_Kr_; hERG trafficking inhibition	^50^
Haloperidol	1967–NA	blocking of DRD1, DRD2, GRIN2B; inverse activation of DRD3	QT prolongation, TdP, sudden cardiac death	inhibition of I_Kr_, I_Na_, I_CaL_	^51^
Levomethadyl acetate	1991–2003	activation of OPRM1	QT prolongation, TdP	inhibition of I_Kr_	^52^
Lidocaine	1944–NA	inhibition of SCN10A, SCN9A, SCN5A; blocking of EGFR	bradycardia, cardiac arrest	inhibition of voltage-gated sodium channel, mitochondrial toxicity	^53^^,^^54^
Methysergide	1965–NA	blocking of HTR2A, HTR2B, HTR2C, HTR7; activation of HTR1A; binding HTR1B, HTR1E, HTR1F	valvular heart disease	induction of fibrosis via HTR2B-induced activation of TGF-β signaling	^6^
Pergolide	1987–NA	blocking of ADRA1B, ADRA2A, ADRA2B, ADRA2C, DRD1, DRD2, DRD3, DRD4, DRD5, HTR1A, HTR1D, HTR2B, HTR2C	valvular heart disease	induction of fibrosis via HTR2B-induced activation of TGF-β signaling	^6^^,^^55^
Phentermine	1959–1997	inhibition of SLC6A2, SLC6A3, SLC6A4; blocking of MAOA, MAOB	valvular heart disease	HTR2B-induced activation of TGF-β signaling	^47^
Propoxyphene	1957–2010	activation of OP1, OP2, OP3	QT prolongation, TdP	inhibition of I_Kr_	^56^
Sertindole	1998^a^–2014	blocking of DRD2, HTR2A, HTR2C, HTR6	QT prolongation, TdP, sudden cardiac death	inhibition of I_Kr_	^57^
Sibutramine	2001–2002	inhibition of SLC6A4, SLC6A2, SLC6A3	myocardial infarction	inhibition of I_Kr_	^58^^,^^59^^,^^60^
Thioridazine	1978–NA	blocking of ADRA1A, ADRA1B, DRD1, DRD2, HTR2A; inhibition of KCNH2	QT prolongation, TdP, sudden cardiac death	inhibition of I_NaL_, I_Kr_	^51^
Venlafaxine	1986–NA	inhibition of SLC6A4, SLC6A2	QT prolongation, arrhythmias	inhibition of I_Na_	^61^^,^^62^
Ziprasidone	2001–NA	inhibition of ADRA1A, ADRA1B, ADRA2A, ADRA2B, ADRA2C, CHRM1, CHRM2, CHRM3, CHRM4, CHRM5, DRD1, DRD2, DRD3, DRD4, DRD5, HTR2A, HTR1B, HTR1D, HTR1E, HTR2C, HTR3A, HTR6, HTR7, HRH1; activation of HTR1A	QT prolongation, TdP, sudden cardiac death	inhibition of I_Kr_	^51^

**Gastrointestinal agents**					

Cisapride	1980–2000	blocking of HTR2A, HTR3A, HTR4; inhibition of KCNH2	ventricular arrhythmia, QT prolongation, TdP, cardiac arrest	inhibition of I_Kr_	^63^
Loperamide	1976–NA	blocking of OPRM1, OPROD1, OPRK; inhibition of POMC; modulation of CALM1	cardiac arrest, QT prolongation, ventricular tachycardia, TdP	inhibition of voltage-gated calcium channels	^64^^,^^65^
Omeprazole	1987–NA	inhibition of ATP4A	acute myocardial infarction, heart failure	disruption of NO synthesis via ADMA production	^66^
Tegaserod	1997–2007^b^	blocking of HTR2A, HTR2B, HTR2C, HTR4	ischemia	inhibition of I_Kr_	^67^

**Genitourinary system agent**					

Terodiline	1975–1991	blocking of muscarinic acetylcholine receptors	ventricular tachycardia, cardiac death	inhibition of I_Kr_, blocking of calcium cycling	^68^^,^^69^

**Antiallergic agents**					

Astemizole	1992–1999	blocking of HRH1, inhibition of KCNH2	long QT syndrome, TdP	inhibition of I_Kr_	^70^^,^^71^
Diphenhydramine	1946–NA	blocking of HRH1, CHRM2	QT prolongation	inhibition of I_Kr_	^72^^,^^73^
Terfenadine	1985–1997	blocking of HRH1	QT prolongation, TdP	inhibition of I_Kr_	^74^

**Anti-infective agents**					

Azidothymidine	1989–NA	inhibition of Pol, TERT	dilated cardiomyopathy	mitochondrial toxicity	^75^
Azithromycin	1988–NA	inhibition of 23S rRNA, rpID, rpIV, PADI4	QT prolongation, TdP, cardiac death	mitochondrial toxicity	^76^
Clarithromycin	1993–NA	inhibition of rpIJ, SLCO1B1, SLCO1B3	QT prolongation, myocardial infarction, arrhythmias, cardiac death	mitochondrial toxicity	^77^^,^^78^
Erythromycin	1955–NA	inhibition of 23S rRNA, MLNR, KCNH2, ALB	QT prolongation, ventricular tachycardia, TdP, ventricular fibrillation	mitochondrial toxicity	^7^
Grepafloxacin	1998–1999	inhibition of gyrA, parC	QT prolongation	inhibition of I_Kr_	^79^
Sofosbuvir	2013–NA	inhibition of NS5b	bradycardia	unknown	^80^
Sparfloxacin	1997–2001	inhibition of parC, gyrA, TOP2A	QT prolongation	inhibition of I_Kr_	^79^
Pentamidine	1975–NA	inhibition of RNA transfer	QT prolongation, arrhythmias	hERG trafficking inhibition	^81^^,^^82^, ^83^, ^84^

**Cardiovascular agents**					

Buflomedil	1970s^c^–2011	blocking of ADRA1A, ADRA2A	QT prolongation, cardiac arrest	unknown	^85^^,^^86^
Dofetilide	2000–2004	inhibition of KCNH2, KCNK2, KCNJ12	QT prolongation, TdP	inhibition of I_Kr_	^87^
Encainide	1987–1991	inhibition of SCN5A	QT prolongation, TdP	inhibition of I_Kr_	^88^
Lidoflazine	1973–1989	blocking of calcium channels	QT prolongation	inhibition of I_Kr_	^89^
Mibefradil	1997–1998	inhibition of CACNA1G, CACNA1H, CACNA1C, CACNA1D, CACNA1F, CACNA1I, CACNA1S, CACNB1, CACNB2, CACNB3, CACNB4	QT prolongation	inhibition of Kir	^90^^,^^91^
Orciprenaline	1965–2011	activation of ADRB2	tachycardia, palpitations	activation of ADRB2	^92^
Prenylamine	1960s–1988	blocking of MYLK2, CALM	QT prolongation, sudden cardiac death, ventricular tachycardia, TdP	inhibition of I_Kr_	^85^
Probucol	1995–2009^d^	inductor of LDL catabolism	QT prolongation, arrhythmias	hERG trafficking inhibition	^82^

**Other agents**					

Alogliptin	2013–NA	inhibition of DPP4	heart failure	unknown	^93^
Clobutinol	1963–2007	inhibition of GABA receptors	QT prolongation	inhibition of I_Kr_	^94^
Rosiglitazone	1994–NA	activation of PPARG	heart failure^e^	unknown	^95^
Saxagliptin	2009–NA	inhibition of DPP4	heart failure	unknown	^96^

TdP, torsade de pointes.

a Australia approved.

b Available only under a restricted distribution program.

c EMA approved only.

d Removed from the US market.

e FDA lifted restrictions for rosiglitazone and confirmed its safety.

## Main molecular mechanisms of drug cardiotoxicity

**Figure 2:**
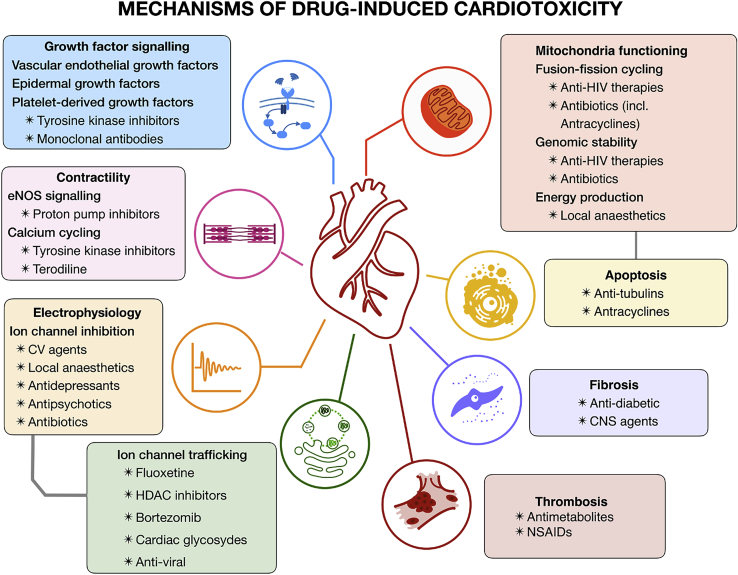
Mechanisms of drug-induced cardiotoxicity Antibiotics and antiviral therapies can induce mitochondrial dysfunction, leading to an impaired fusion-fission cycle. Local anesthetics increase mitochondrial permeability, affecting function. Cardiotoxicity of tyrosine kinase inhibitors and monoclonal antibodies is primarily linked to inhibition of major signaling pathways for cardiomyocyte survival and maintenance. Different CNS and antidiabetic agents have been associated with the emergence of cardiac fibrosis. Proton pump inhibitors can affect cardiac contractility via inhibition of NO synthesis. Similarly, tyrosine kinases inhibitors have been linked to calcium cycling dysregulation. Multiple neural, cardiovascular, and anti-infective agents also have been shown to interact with cardiac electrophysiology. Equivalently, cardiac adverse effects of fluoxetine, HDAC inhibitors, and bortezomib are primarily linked to affecting ion channel trafficking rather than acute channel block. CV, cardiovascular; NSAIDs, nonsteroidal anti-inflammatory drugs; HDAC, histone deacetylase; eNOS, endothelial nitric oxide synthase.

**Figure 3:**
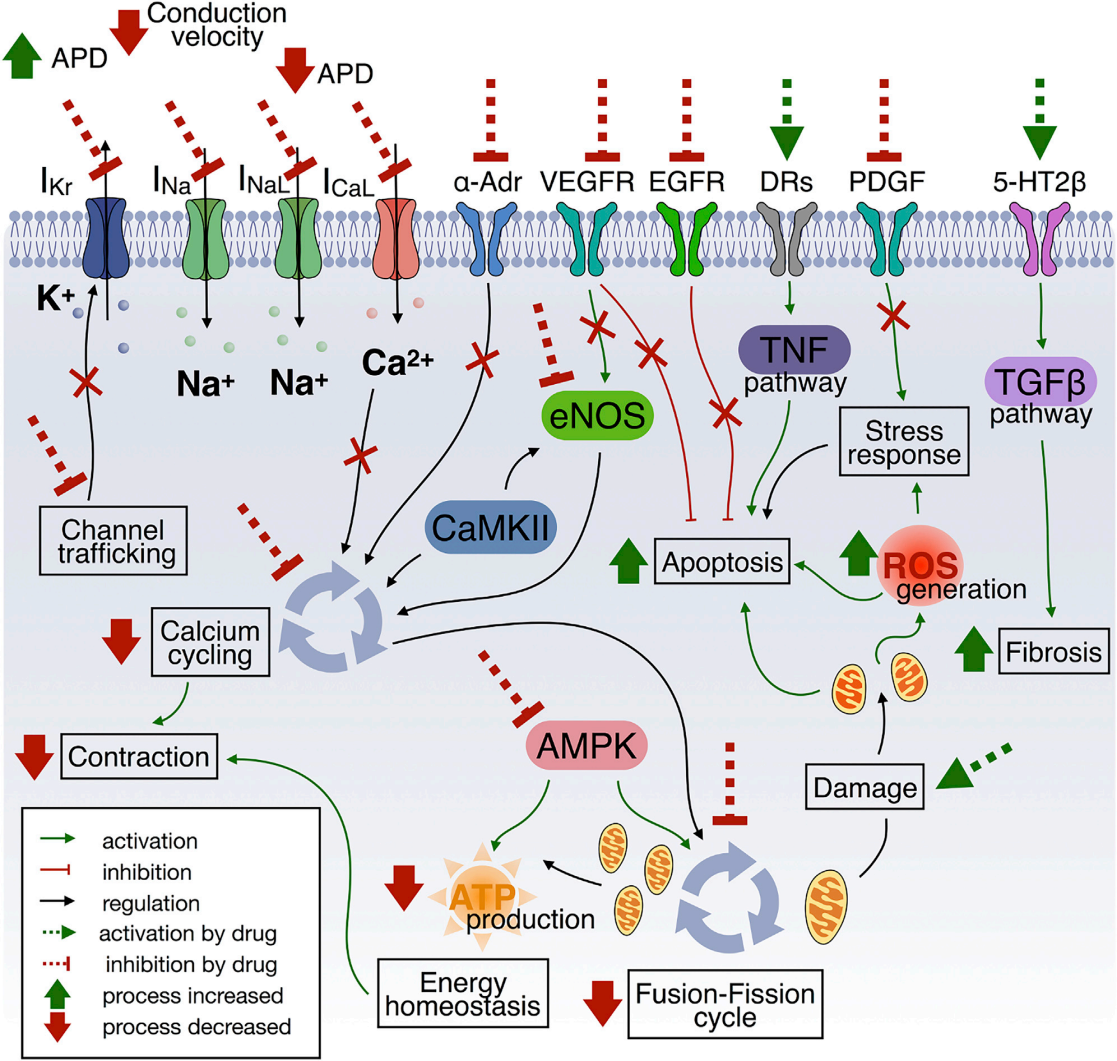
Overview of mechanisms of cardiotoxicity Abnormalities in action potential duration or conduction velocity are associated with a direct block of ionic currents or inhibition of their trafficking from nucleus to cell membrane. Multiple pathways can trigger apoptosis, including VEGFR or EGFR inhibition (cardiomyocyte survival), PDGF inhibition (compensatory stress response), DR-induced TNF signaling activation, mitochondrial damage, or elevated ROS levels. α-Adr, VEGFR signaling, or eNOS inhibition affects calcium cycling. AMPK signaling inhibition affects both the mitochondrial fusion-fission cycle and the production of ATP. Serotonin-induced activation of the TGF-β pathway is primarily linked to cardiac fibrosis induction. I_Kr_, rapid delayed rectifier current; I_Na_, inward sodium current; I_NaL_, late inward sodium current; I_CaL_, L-type calcium current; α-Adr, alpha-adrenergic receptor; VEGFR, vascular endothelial growth factor receptor; EGFR, epidermal growth factor receptor; DRs, death receptors; PDGF, platelet-derived growth factor; 5-HT2β, 5-hydroxytryptamine receptor (serotonin receptor 2B); TNF, tumor necrosis factor; TGF-β, transforming growth factor beta; ROS, reactive oxygen species; CaMKII, calcium/calmodulin-dependent protein kinase II; AMPK, AMP-activated protein kinase; ATP, adenosine triphosphate.

Cardiotoxicity generally results from the simultaneous interruption of key myocardial functions and viabilities. In this section, we focus on adverse drug effects via the disruption of electrophysiology, contractility, mitochondrial toxicity, growth factor, and cytokine regulation. These mechanisms of drug cardiotoxicity are summarized in Figure 2, together with the main types of therapeutic agents manifesting such risks. Importantly, the mechanisms underlying adverse cardiac events are often multifactorial, exerting their action through complex cell signaling pathways. The main signaling pathways underlying drug cardiotoxicity, further developed in the subsequent sections of this review paper, are illustrated in Figure 3.

### Electrophysiology

#### Direct block

hERG-encoded channels, carrying the rapid delayed rectifier current (I_Kr_), are main determinants of cardiac repolarization and proarrhythmic events (Figure 3, top left). Therefore, in the last decades, preclinical testing for cardiac adverse events has primarily focused on screening assays of hERG channel inhibition.^97^ However, the biological role of hERG channels is not limited to their electrophysiological function. hERG-encoded channels are also involved in cell proliferation and malignant cell apoptosis,^98^ and reducing hERG expression in gliomas has been proposed as a target for antineoplastic therapy.^99^ Beyond I_Kr_ block, drugs often interact with multiple channels. Recent work on multichannel action and its connection to adverse effects has resulted in significantly improved toxicity prediction.^100^^,^^101^ Evidence also suggests that the electrophysiological implications of drug-induced block of hERG channels could be alleviated by interactions with other channels.^102^ Adverse electrophysiological effects can be accentuated by several factors, especially heart disease. For example, diabetes has been shown to abate the amplitude of multiple ionic currents,^103^ enhancing susceptibility to adverse effects.

#### Trafficking inhibition

**Figure 4:**
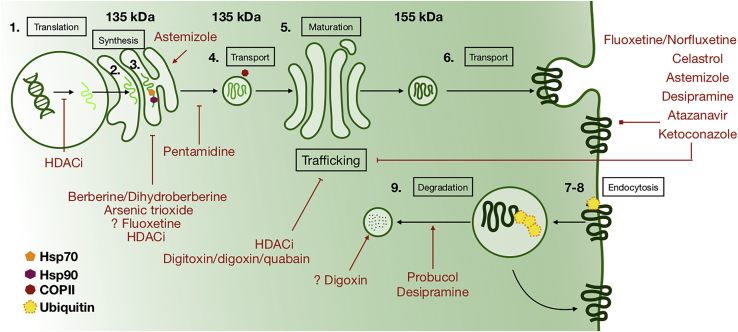
Schematic of hERG biogenesis, trafficking, and degradation and pathways of drug-induced I_Kr_ deficiency Subcellular processes modulating hERG expression on the cellular membrane include (1) mRNA synthesis, (2) synthesis of polypeptide chain, (3) transport of polypeptide from endoplasmic reticulum to Golgi apparatus with Hsp70 and Hsp90 chaperones attached, (4) transport of the polypeptide in the COPII-mediated vesicle, (5) glycosylation, (6) exocytosis, (7) endocytosis, (8) ubiquitination, and (9) degradation of hERG.

In addition to direct hERG channel block, multiple pharmacological agents can cause hERG deficiency (with hERG channel block or independently) by the inhibition of its biogenesis and trafficking. A detailed schematic of the subcellular processes involved in hERG biogenesis, trafficking, and degradation, together with established pathways of drug-induced I_Kr_ deficiency, is presented in Figure 4. Drugs with reported effects on hERG trafficking impairment are listed in Table 1. Compared with the fast action of acute inhibition, hERG trafficking impairment manifests with timescales of hours to days.

For example, arsenic trioxide (Figure 4), extensively used in cancer treatment, interacts with chaperones Hsp70 and Hsp90, altering the folding process of hERG. As a result, it has been shown to affect hERG maturation in HEK293 cell lines.^104^ Folding inhibition also has been proposed as a mechanism of the dual tumor suppression and carcinogenicity of arsenic trioxide.^105^ Similarly, the antidiarrheal berberine and its derivative, dihydroberberine (Figure 4), have been shown to impair hERG folding in both HEK293 cell lines and guinea pig ventricular cardiomyocytes.^106^^,^^107^ The inhibition of folding by those drugs results in the reduction of the mature glycosylated form of 155 kDa hERG, which can be easily measured along with immature hERG (135 kDa). Accumulation of unfolded proteins activates the unfolded protein response pathway, as shown in HEK293 cells incubated with berberine^107^ and in mouse vascular endothelium cell lines incubated with arsenic trioxide.^108^ For the anti-infective drug pentamidine (Figure 4), mature hERG deficiency was identified as the result of reduced immature transport in HEK293 cell lines and neonatal rat cardiomyocytes.^81^

Cardiac glycosides (Figure 4), specifically Na^+^/K^+^-ATPase inhibitors with known QT prolongation risk, reduce hERG trafficking in HEK293 lines and in guinea pig ventricular cardiomyocytes.^109^ Incubation with digitoxin, digoxin, and ouabain resulted in a significant reduction of mature hERG. Later evidence showed that digoxin-induced hERG trafficking inhibition is a result of the low intracellular potassium concentration caused by Na^+^/K^+^-ATPase inhibition.^110^ Moreover, digoxin has been reported to generally enhance protein degradation through a lysosomal pathway.^111^

The lipid-lowering compound probucol also causes QT prolongation without a direct block of hERG channels (Figure 4). This is believed to result from trafficking disruption, as shown in neonatal rat ventricular cardiomyocytes^112^ and later in HEK293 cells, in which enhanced degradation of mature hERG via neddylation was indicated as a mechanism.^113^ Meanwhile, arsenic trioxide showed no effect on neddylation of hERG.^113^ Gene expression inhibition and alteration of the acetylation of proteins involved in ion channel trafficking and degradation also have been proposed as possible cardiotoxic mechanisms of histone deacetylase (HDAC) inhibitors.^114^

In addition, a substantial number of drugs are known to bind to the hERG channel, simultaneously inhibiting it and disrupting trafficking of the mature channel. One example is ketoconazole (Figure 4), an anti-infective agent that causes clinical QT prolongation and has been shown to co-inhibit I_Kr_ and hERG trafficking in HEK293 cells.^115^ Interestingly, reduced mature hERG was observed in both wild-type and nonbinding mutant channels, suggesting trafficking impairment as main mechanism. However, this was not studied in detail, and biological assays showed inconsistent results with inhibition of the Hsp90 chaperone in yeast strains, but not in cancer or rabbit cell lysate Hsp90. Similar effects in HEK293 cells were shown by atazanavir, another anti-infective agent with known arrhythmic risk,^116^ and by the antidepressants fluoxetine and norfluoxetine.^117^ In contrast, the inhibition and trafficking mechanisms of the antidepressant desipramine (Figure 4) have been well characterized.^118^ Like probucol, desipramine accelerates degradation of mature hERG in HEK293 cells without interfering with Hsp90 and Hsp70 chaperones. A study of celastrol in HEK293 cells expressing hERG, KCNJ2, and KCNB1 genes showed it inhibits both the function and the trafficking of K_ir_, encoded by KCNJ2, and hERG channels, but not K_v_2.1, encoded by KCNB1.^119^ hERG trafficking effects were 5- to 10-fold more potent than the acute block of the channel.

### Contractility

Drugs have also been observed to affect contractility, the most fundamental heart function. Cardiomyocytes connect electromechanically with one another and the extracellular matrix via gap junctions, key molecular regulators for intercellular communication and coordinated contraction. In addition, calcium cycling, a complex process involving multiple regulatory molecules, is essential for contraction.

Specifically, the calcium/calmodulin-dependent protein kinase II (CaMKII) regulates calcium cycling via phosphorylation of its targets on both the cellular membrane and the sarcoplasmic reticulum (Figure 3, center). Accordingly, CaMKII inhibition has been shown to have potential efficacy for antiarrhythmic therapy.^120^ Nitric oxide (NO) further regulates intracellular calcium, promotes vascular relaxation, and inhibits platelet aggregation. In cardiomyocytes, NO is primarily synthesized by endothelial nitric oxide synthase (eNOS), in turn regulated by CaMKII. Protein phosphatases, which act in opposition to CaMKII, are believed to regulate cardiac gap junction communication.^121^

Depressing either contractility or heart rate is a mechanism of action of many cardiac drugs, such as calcium-channel blockers that bind to L-type calcium channels and beta blockers that block beta adrenoceptors. Cardiac glycosides, which inhibit the Na^+^/K^+^ pump, can also depress heart rate, despite their well-known positive inotropic effects. The safe administration of cardiac glycosides is regarded as a difficult task because of their narrow safety margins,^122^ and the most potent inotropic agents generally have the lowest toxic-to-therapeutic ratios.^123^

However, some medications have been reported to disrupt contractility on other levels. The destabilization of the homeostatic calcium system has been proposed as the cardiotoxic mechanism of proton pump inhibitors. These have been suggested to inhibit dimethylarginine dimethylaminohydrolase (DDAH), an enzyme responsible for eliminating asymmetric dimethylarginine (ADMA), resulting in excess of ADMA impeding NO synthesis.^124^ Similarly, antineoplastic agents such as tyrosine kinases have been linked to upregulated CaMKII expression and activity.^125^

### Mitochondrial toxicity

Mitochondria play an important role in cardiac function, mainly by satisfying the immense energy requirements of contraction. As such, failure to replace malfunctioning mitochondria is highly injurious. Altered cardiac metabolism has been linked to cardiac disease development, such as ischemia,^126^ and conditions that increase the risk thereof, such as diabetes.^127^ Further demonstrating their association, contractility and the mitochondrial fusion-fission system were recently shown to be closely coupled (Figure 3): the fusion-fission cycle depends on calcium homeostasis, and fusion-fission abnormalities can lead to aberrant contraction.^128^

As a by-product of their metabolic function, mitochondria produce reactive oxygen species (ROS) that, as shown in Figure 3, play an important role in proapoptotic signaling.^129^ ROS also engage dynamically in the mitochondrial fusion-fission cycle to respond to environmental stressors. Although mitochondrial fusion and fission help to reduce cellular stress under mild environmental stressors, these processes can also result in apoptosis and tissue necrosis in response to extreme stressors.^130^

Several pharmaceutical agents have been found to produce or facilitate mitochondrial toxicity, including in the heart.^131^ These inimical drugs span multiple classes, including anthracyclines, antivirals, antidepressants, and local anesthetics. The mitochondrial toxicity of anthracyclines is primarily linked to the inhibition of their direct target, topoisomerase (DNA) II beta (TOP2B), required for mitochondrial DNA replication.^132^ Antiretroviral drugs, often used to treat HIV, directly target and inhibit reverse transcriptase. However, antiretrovirals have been found to also inhibit mitochondrial DNA polymerase gamma (DNA Pol-γ), depleting mitochondrial DNA and disrupting mitochondrial function.^133^ Moreover, anti-HIV therapies have also been shown to inhibit the mitochondrial fusion-fission cycle.^8^ Local anesthetics have been suggested to interact with phospholipids on the mitochondrial membrane, which often result in increased membrane permeability, electron transport chain disruption, and calcium accumulation.^134^

### Growth factors and cytokine signaling

Growth factors and cytokines are biologically active molecules that act directly on many cellular functions, such as adhesion, proliferation, and migration. Consequently, growth factor and cytokine signaling broadly affect tissue and organ function (Figure 3, top right).

Vascular endothelial growth factor (VEGF) is a signal protein responsible for the regulation of vascular formation, angiogenesis, cardiomyocyte development and proliferation, and myocardial regeneration. VEGF is also a major antiapoptotic factor that promotes cardiomyocyte survival in response to environmental stress or disease.^135^^,^^136^ Because angiogenesis is closely related to neoplastic metastasis and malignancy, controlling angiogenesis via inhibition of VEGF signaling is an attractive target.^137^ However, VEGF-inhibitory therapies are known to produce many adverse side effects, including cardiotoxicity.^138^ Closely related to VEGF, the platelet-derived growth factor (PDGF) is also key in myocardial development,^139^ in the angiogenic cardiovascular compensatory stress response,^140^ and in cardiac fibrosis.^141^ Inhibition of PDGF by sunitinib has been linked to its cardiotoxicity.^138^

The epidermal growth factor receptor (EGFR) belongs to the epidermal growth factor (EGF) family of protein kinases, whose members include HER2, HER3, and HER4, and is essential to almost every known cellular process. HER2 is particularly crucial for cardiomyocyte differentiation and embryonic cardiac development.^142^ EGFR signaling is antiapoptotic, indicating it could lead to uncontrolled growth or intensified oncogenesis. Sufficient evidence suggests that EGF-activated VEGF signaling may enable the regulation of both angiogenesis and eNOS signaling.^143^ Taking these factors together, targeting EGFR to inhibit neoplastic growth has become an attractive avenue for potential antineoplastic therapies. Unfortunately, EGF inhibitors, such as lapatinib or trastuzumab, have recently been linked to adverse cardiac effects.^33^ Activation of AMP-activated protein kinase (AMPK), which controls cellular energy and survival homeostasis and is required for mitochondrial fission in response to stress,^144^ could underlie the relatively low toxicity rate of lapatinib^33^ compared with the myocardial metabolism disruption associated to AMPK attenuation by other tyrosine kinase inhibitors.^138^

## Main classes of drugs causing clinical cardiotoxicity

### Antineoplastic agents

Although significant improvements have arisen in recent years in chemotherapy safety, cardiovascular damage remains one of the most common adverse side effects of antineoplastic agents, often resulting in diminished life expectancy for cancer patients. Two types of chemotherapy-induced cardiotoxicity have been established: irreversible (type I) and reversible (type II), respectively characterized by cellular damage and dysfunction.

#### Antimitotics

##### Anthracyclines

Anthracyclines have been shown to be effective for the widest range of antineoplastic applications. They have four mechanisms of action by which they target actively proliferating neoplastic cells: DNA-RNA intercalation, TOP2B inhibition, iron-mediated generation of free radicals, and induction of the eviction of histone from chromatin.^24^

The cumulative and dose-related cardiotoxicities of anthracyclines are primarily linked to ROS formation, mitochondrial dysfunction, and cardiomyocyte apoptosis^145^ (Figure 3). Doxorubicin, a member of this class of agents, also has been shown to upregulate the expression of tumor necrosis factor (TNF)-mediated death receptors, involved in apoptosis in cardiomyocytes derived from induced pluripotent stem cells.^5^ Although cumulative cardiotoxicity is frequent among anthracyclines, the manufacturers of several claim no evidence of induced cardiotoxicity. For example, this is the case with amrubicin, approved by the Food and Drug Administration (FDA) of the United States of America, and marketed as Calsed in 2011 for lung cancer treatment.

##### Antimetabolites

Antimetabolites suppress the division of potentially cancerous, quickly dividing cells via interference with DNA replication. Because of their mechanism of action, their most frequent side effects are observed in highly proliferative tissues, such as the gastrointestinal tract, skin, or hair. Although less common, if cardiotoxicity arises, it generally does so within a week, with severity ranging from symptomatic arrhythmias to sudden cardiac death.^9^ The proposed mechanisms include multifactorial effects on the cardiovascular system: for example, 5-fluorouracil and cisplatin activate thromboxane A2 (TXA_2_) formation and platelet aggregation, potentially leading to cardiac remodeling and ischemia.^146^^,^^147^ Both drugs are also known to cause electrolyte imbalances, which play an important role in gastrointestinal toxicity,^148^ and are believed to be involved in their cardiotoxicity.^149^ Cytarabine is another antimetabolite with known cardiotoxicity;^17^^,^^18^ however, an understanding of its cardiotoxic mechanisms is still lacking.

##### Antitubulins

*Vinca* alkaloids and taxanes eliminate tumors by binding to microtubules. Like anthracyclines and antimetabolites, they target cell division and are primarily linked to gastrointestinal toxicity, although several antitubulins have been connected to specific adverse cardiac events. For example, this is the case with vinblastine, additionally associated with myocardial ischemia and infarction.^36^ Similarly, paclitaxel and docetaxel have been linked with bradycardia, ischemia, and heart failure.^22^ One of their proposed cardiotoxic mechanisms involves the inhibition of actively proliferating cardiac endothelial cells.^150^ However, given the sequential or combinatorial nature of cancer treatment, tubulin inhibitors are frequently administered after anthracyclines, which are often involved in cardiovascular adverse events. Therefore, the precise cardiotoxic role of tubulin inhibitors remains open for debate.

#### Tyrosine kinase inhibitors

Rather than targeting proliferating tissues, tyrosine kinase inhibitors exert their action by inhibiting tyrosine kinases as main enzymes responsible for the activation of signaling cascades in the synthesis of proteins. Despite reduced rates of side toxicity, some tyrosine kinase inhibitors have been associated with cardiovascular system damage. Cardiotoxicity is primarily linked to inhibition of major signaling pathways responsible for cardiomyocyte survival and maintenance, such as in the case of sorafenib or vandetanib, which are VEGF signaling inhibitors.^138^ However, lapatinib, an EGFR signaling inhibitor, presents low cardiotoxicity rates. As previously mentioned, this presumably results from its activation of the AMPK signaling pathway (Figure 3), which mobilizes cardiomyocytes and increases ATP synthesis and storage.^33^ In contrast, the tyrosine kinase inhibitor sunitinib, whose targets include vascular endothelial growth factor receptor (VEGFR), also inhibits the AMPK signaling pathway and potentially inhibits energy metabolism and PDGF signaling (Figure 3), all involved in the cardiomyocyte mechanical stress response.^138^^,^^32^ Different cardiotoxic mechanisms have been proposed for other tyrosine kinase inhibitors. Imatinib inhibits the chimeric oncogene bcr-abl, the protein constructed by the fusion of a breakpoint cluster region (bcr) with an Abelson tyrosine kinase (abl). Its cardiotoxicity is primarily linked to the release of B cell lymphoma 2 (Bcl-2) proteins, which cause mitochondrial damage.^138^ Interestingly, imatinib can inhibit the proliferation and apoptosis of neoplastic cells via hERG inhibition. Sunitinib and imatinib have demonstrated the ability to activate CaMKII expression and activity *in vitro* but without affecting myocardial contractility.^125^ In addition, sunitinib and imatinib induce high ROS levels (see also Figure 3), leading to reduced cell viability.^151^ Other bcr-abl inhibitors, such as dasatinib and nilotinib, are also known to induce cardiac adverse events,^21^ presumably linked to VEGF signaling inhibition.^152^

#### Monoclonal antibodies

Monoclonal antibodies, identical to antibodies produced by the immune system, specifically bind to extracellular and cell surface proteins, activating cellular apoptosis and blocking tumor proliferation. The adverse effects of this class of agents are primarily linked to their targets. HER2 inhibition by monoclonal antibodies is principally associated with cardiac dysfunction.^33^ Trastuzumab, a vascular endothelial growth factor A (VEGFA) inhibitor, is also known to downregulate Neuregulin-1, a signaling molecule in cardiac homeostasis and development.^34^ Its most common side effect is hypertension, but myocardial infarction may also occur.^13^ Bevacizumab, another monoclonal antibody, also negatively affects the coagulation system, probably because of its VEGFR inhibition effects.^13^

#### HDAC inhibitors

HDACs are a class of enzymes that remove acetyl groups from an amino acid on a histone. HDAC inhibitors, used for neurological disease, have recently been proposed as a powerful new class of antineoplastic agents. However, growing concern regarding their cardiac safety has slowed their progress into clinical trials. For example, romidepsin, approved for clinical use in 2009, has been suggested to produce diverse cardiac adverse effects, including QT prolongation, torsade de pointes arrhythmias, and sudden cardiac death.^153^ Its cardiac adverse events are primarily linked to hERG trafficking inhibition rather than direct channel block.^114^ To date, 17 HDAC inhibitors exist, classically divided into 4 classes. However, cardiotoxicity is not class specific. HDAC inhibitors of classes I, II, and IV are generally used as antineoplastics, whereas class III (known as sirtuins) could potentially be used as a cardioprotective agent by mainly reducing the risk of thrombosis, atherosclerosis, and endothelial dysfunction.^154^

#### Other antineoplastic agents

Bortezomib is a proteasome inhibitor approved for myeloma treatment that inhibits the ability of malignant cells to escape apoptosis. Potential side effects include neutropenia, thrombocytopenia, and cardiotoxicity. Cardiac adverse events are considered reversible; however, bortezomib has been reported to cause arrhythmias and to even lead to heart failure.^14^^,^^15^ Cardiotoxicity is linked to its primary target, the ubiquitin-proteasome system, which is essential for cardiomyocyte and mitochondria function and controls hERG trafficking.^155^

Recent reports have also raised concerns over the cardiac safety of immune checkpoint inhibitors, newer and highly effective anticancer therapies.^25^^,^^26^ Given their target, checkpoint inhibitors have been mainly associated with immune-related complications, such in the case of vitiligo.^156^ Lethal myocarditis has also been reported, although the underlying cardiotoxic mechanism remains unknown.

Used in leukemia, arsenic trioxide is another example of an anticancer drug with undesired cardiac complications, such as ventricular and supraventricular tachycardias.^11^^,^^12^ Its hERG trafficking inhibition (Figure 4) results in I_Kr_ deficiency and action potential prolongation in both animal and human ventricular cells.^157^

### CNS agents

The electrophysiological function of both myocardial and nervous tissues requires the propagation of action potentials, a process triggered by external and intracellular mechanisms and involving the depolarization and repolarization of cellular membranes. Given the similarities in electrophysiology, CNS agents that target neuronal electrophysiology can also affect cardiac tissue. Consequently, drugs that cause neurotoxicity may also result in cardiotoxicity, although neurotoxicity is typically exhibited at lower doses.^158^ Other CNS agents have alternative mechanisms of cardiotoxicity that do not involve alterations of the electrophysiology.

#### Local anesthetics

Both heart and nervous tissues depend on sodium channels for action potential triggering. Local anesthetics frequently block sodium channels (Figure 3, top left) and therefore directly affecting both systems. The most cardiotoxic and neurotoxic local anesthetic is cocaine, first used in 1884.^45^ The next generation of local anesthetics, such a bupivacaine and lidocaine, was intentionally developed to overcome this toxicity. However, clinical evaluations demonstrated that bupivacaine (markedly more cardiotoxic than other local anesthetics) causes hemodynamic and electrophysiological disturbance, which may result in hypoxia, arrhythmia, and even cardiac arrest. Lidocaine and ropivacaine have also been shown to cause dose-dependent adverse cardiac reactions.^41^^,^^53^ A hypothesized mechanism of cardiotoxicity involves interactions between local anesthetics and mitochondrial membranes (Figure 3) by increasing the permeability of cardiolipin, thus interrupting function and potentially inducing apoptosis.^134^

#### Antidepressants

Available evidence emphasizes antidepressants as a CNS class with significant cardiovascular side effects. Indeed, several antidepressants were withdrawn from the market or restricted for use because of cardiac adverse reactions. Tricyclic antidepressants and neuroleptics directly interact with sodium, calcium, and potassium channels (Figure 3, top left) and can cause fatal arrhythmias and hypotension in overdose.^159^ The next generation of antidepressants is considered of lower risk, with fewer cardiac adverse effects reported, but they too inhibit cardiac sodium, calcium, and potassium channels.^160^ As an example, the selective serotonin inhibitor fluoxetine inhibits the L-type calcium current (I_CaL_) and I_Kr_ in addition to its primary targets. It has also been shown to disrupt hERG trafficking,^117^ and long-term use may result in mild bradycardia. The potentially more arrhythmic venlafaxine, another selective serotonin inhibitor, has been shown to block the inward sodium current (I_Na_) in both humans and animals.^72^^,^^161^

#### Antipsychotics

Psychiatric disorders are known to be accompanied by an increased risk of cardiovascular disease.^162^ In addition, many antipsychotics have been found to cause QT prolongation and cardiotoxicity.^57^ Clozapine, a highly effective treatment for schizophrenia, has been linked to serious complications such as myocarditis, an inflammatory cardiac muscle disease.^43^^,^^44^ Therefore, it is generally considered the last resort if other antipsychotics are ineffective. However, the mechanism underlying clozapine-induced myocarditis is unknown. The antipsychotic sertindole was removed from market in 1998 due to QT prolongation and sudden cardiac death, primarily because of its high-affinity I_Kr_ block.^163^ However, it was relaunched on the European market in 2005 with required cardiac monitoring. Other antipsychotics for schizophrenia and bipolar disorder, such as thioridazine, ziprasidone, and haloperidol, can lead to QT prolongation, torsade de pointes arrhythmias, or even sudden cardiac death.^51^ Ziprasidone has been shown to block I_Kr_, thioridazine blocks both late inward sodium current (I_NaL_) and I_Kr_, and haloperidol blocks I_Kr_, I_Na_, and I_CaL_ without affecting I_NaL_.^160^^,^^164^ Ziprasidone and clozapine also have been found to block alpha-adrenergic receptors,^165^ involved in calcium cycling regulation in cardiomyocytes.

#### Neurodegenerative disease agents

At present, neurodegenerative diseases such as Alzheimer’s disease and Parkinson’s disease are incurable, attracting immense interest in the development of prospective therapies. Among them is pergolide, a dopamine receptor antagonist with potential application in Parkinson’s disease. However, pergolide has been associated with the emergence of fibrosis in multiple tissues, including the heart.^6^ This is believed to stem from pergolide activating serotonin HTR2B receptors (Figure 3, right), inducing the transforming growth factor beta (TGF-β) pathway.^55^^,^^166^ Pergolide use was consequently restricted because of increased risk of fibrotic valvular heart disease.

#### Other CNS agents

Further applications of CNS agents include their use in pain or appetite control. Methysergide and ergotamine, serotonin 5-hydroxytryptamine (5-HT) receptor inhibitors (Figure 3, right side) used for migraines, are known to share adverse cardiac effects with pergolide and to cause cardiac fibrosis.^48^ In addition, several appetite suppressants, including dexfenfluramine, fenfluramine and phentermine, chlorphentermine, and benfluorex, were lifted from the market due to cardiac fibrosis. The primary mechanism of fibrosis induction is believed to involve the activation of the TGF-β pathway through 5-HT_2B_ receptors. Another discontinued appetite suppressant drug, sibutramine, was linked to myocardial infarction. Its underlying mechanism remains undescribed, but *in vitro* experiments showed that sibutramine blocks the I_Kr_ channel.^58^

Opioid analgesics also have been shown to affect cardiac electrophysiology.^56^ This led to the withdrawal of levomethadyl acetate in 2001 from the European market and propoxyphene in 2010. Although the FDA reported that levomethadyl acetate had no safety concerns, it was also lifted from the US market in 2003.

### Genitourinary system agents

Muscarinic receptor antagonists are a class of drugs approved for treatment of incontinence. Although generally considered safe, they have a well-known potential to affect cardiac function and may increase heart rate, prolong QT, and increase cardiovascular risk when used concomitantly with other medications.^167^ Especially relevant within this class, terodiline, a muscarinic receptor antagonist, was withdrawn from the market due to serious cardiac side effects, such as torsade de pointes and even cardiac arrest among older people.^68^ The toxicity of terodiline is linked to the inhibition of the hERG-encoded channel and the disruption of calcium cycling, with its cardiotoxic effects depending on concentration and genetic background.^2^ The withdrawal of terodiline has since raised concerns about potential cardiac adverse reactions by the whole group of muscarinic receptor antagonists.

### Gastrointestinal agents

As in the heart and nervous tissues, the function of the gastrointestinal system is heavily controlled by cellular electrophysiology. 5-HT_4_ receptors, located in the alimentary tract, heart, or CNS, can be pharmacologically modulated to control the release of neurotransmitters. Cisapride, a drug that increases motility in the upper gastrointestinal tract, was designed to block serotonin 5-HT_4_, but it also inhibits hERG, in addition to 5-HT_2A_ and 5-HT_3A_ serotonin receptors.^168^ Its marked hERG inhibition produced cardiac arrhythmias and torsade de pointes, leading to its withdrawal from the market in most countries. Tegaserod, a nonselective 5-HT_4_ inhibitor, was also withdrawn by the FDA in 2007 despite absence of reports showing hERG channel inhibition.^67^ New versions of selective 5-HT_4_ blockers, such as clebopride and mosapride, are considered cardiac safe.^169^

Other types of receptors are also present in the gastrointestinal system. For example, loperamide, an opioid antagonist approved for treating diarrhea symptoms, blocks the mu-opioid receptor. Loperamide has also been shown to inhibit voltage-gated calcium channels and may produce cardiotoxic effects in humans.^64^^,^^68^ In 2016, the FDA issued an announcement indicating that high doses of loperamide, or its misuse, could cause serious cardiac adverse events, including lethal heart attacks.

Proton pump inhibitors, such as omeprazole, are another class of gastrointestinal agents labeled as cardiotoxic. Although a direct connection between omeprazole and cardiotoxicity has never been fully established and the mechanism of action remains unknown,^66^ it may arise because of ADMA production, which interferes with NO synthesis (Figure 3) and accelerates endothelial cellular senescence.^124^^,^^170^

### Antihistamines

H1 antihistamines reduce allergic responses by blocking histamine H1 receptors on the cell surface. The biological role of histamines includes participation in the immune response to pathogens and allergens by increasing capillary permeability. Within the cardiovascular system, the stimulation of H1 receptors results in the constriction of coronary blood vessels, inducing a positive chronotropic effect. However, two second-generation antihistamines, terfenadine and astemizole, were withdrawn from the market due to QT prolongation and torsade de pointes linked to their direct block of I_Kr_.^70^ Diphenhydramine, the first antihistamine introduced to the market, also demonstrated a QT prolongation signature upon overdose.^73^

### Anti-inflammatory agents

Nonsteroidal anti-inflammatory drugs (NSAIDs) are a broad class of agents that target one or both prostaglandin synthases (COX-1 and COX-2) and hence display anti-inflammatory properties. NSAID side toxicities, such as gastrointestinal toxicity, primarily result from COX-1 inhibition, and new generations of NSAIDs selectively target COX-2. However, both selective COX-2 inhibitors and high doses of nonselective COX inhibitors can display cardiotoxic side effects. In 2004, the FDA withdrew the COX-2 inhibitor rofecoxib, one of the most widely used drugs ever withdrawn from the market. Although its significant cardiotoxicity seems to be linked to its unique metabolism,^171^ in 2007, the FDA issued a nonapproval letter for the selective COX-2 inhibitor etoricoxib due to cardiotoxic concerns. In response to these concerns, the FDA strengthened the safety warning on NSAID labels in 2015. Long-term administration of both selective COX-2 inhibitors, such as diclofenac, and nonselective COX-1/COX-2 inhibitors, such as ibuprofen, naproxen, and indomethacin, has been shown to increase the risk of cardiac arrest.^37^ The cardiotoxic mechanism of selective COX-2 inhibitors is primarily linked to blocking prostacyclin synthase without affecting TXA_2_ synthesis, leading to an increased risk of thrombosis.^172^ In contrast to antimetabolites that induce TXA_2_ formation, increasing the risk of ischemia, evidence suggests that COX-2 inhibitors decrease the risk of ischemia because of their ability to reduce inflammation.^173^

### Anti-infective agents

Anti-infective agents are compounds with selective toxicity against pathogens, such as bacteria, viruses, or other microorganisms. Anti-infective agents vary by the mechanism of action and side effects.

#### Antibiotics

Antibiotics, among the most prescribed drugs worldwide, primarily disrupt the bioactive processes of pathogens, such as cell wall construction, protein and nucleic acid metabolism, and repair. Because human cells share some functions with prokaryotic pathogens, some antibiotics (such as anthracyclines) are also used as antineoplastic agents. The cardiotoxicity of cytostatic antibiotic agents has been discussed. Macrolides, which target bacterial protein synthesis via inhibition of prokaryotic ribosomal subunits and are widely used for respiratory infections, have also been shown to block hERG.^174^ Macrolides such as erythromycin, azithromycin, and clarithromycin are considered arrhythmogenic.^7^ It has also been shown that macrolides cause mitochondrial toxicity (Figure 3) by inhibiting protein synthesis in mitochondria.^175^ Fluoroquinolones are another group of antibiotics that can affect hERG and cause torsade de pointes.^79^ Because of this effect, use of fluoroquinolones such as grepafloxacin and sparfloxacin was discontinued.

#### Antivirals

The design of safe antivirals is complicated, because viruses use host cell structures to replicate. A major concern of antiviral therapy is mitochondrial toxicity in liver, skeletal muscle, and heart tissues.^133^ Azidothymidine, an antiretroviral HIV treatment, has been shown to induce mitochondrial dysfunction, leading to mitochondrial fragmentation and an impaired fusion-fission cycle^8^ (Figure 3). Azidothymidine inhibits mitochondrial DNA polymerase alongside its main target, reverse transcriptase.^133^ Sofosbuvir, recently reported as cardiotoxic, is an inhibitor of RNA polymerase nonstructural protein 5B used to treat hepatitis C. Although the drug exhibited a cardiac-safe profile during clinical trials, several cases of severe bradycardia were reported post-marketing.^80^ The mechanism by which sofosbuvir induces cardiac adverse effects is still unclear.

#### Other anti-infective agents

Pentamidine, used to treat leishmaniasis, trypanosomiasis, and pneumonia, has been associated with QT prolongation and ventricular arrhythmias in intravenous treatment.^82^ *In vitro* experiments showed that despite being a poor direct blocker of I_Kr_ at therapeutic concentrations,^83^ pentamidine inhibits hERG trafficking (Figure 4), causing action potential prolongation in both animal and human cells.^81^^,^^84^

### Cardiovascular agents

Paradoxically, the worsening of arrhythmic risk is a serious side effects of some antiarrhythmic agents. Several highly potent drugs were lifted from the market or restricted in use because of proarrhythmic effects, including dofetilide and encainide. Some nonselective calcium blockers, such as lidoflazine and prenylamine, were also suspended because of their life-threatening QT prolongation. This side effect is primarily linked to their high-affinity block of hERG channels.^176^ Buflomedil, an alpha-adrenoceptor antagonist, was withdrawn from the European market due to its unfavorable cardiac safety profile. The selective beta-adrenoceptor blocker orciprenaline was removed from the market due to life-threatening cardiac side effects.^92^

The efficacy and safety of cardiovascular agents have been investigated by landmark clinical trials, such as the Cardiac Arrhythmia Suppression Trial (CAST) focused on the class I antiarrhythmic agents encainide and flecainide.^88^ This study reported increased mortality rates caused by arrhythmia and myocardial infarction shock in patients treated with flecainide and encainide compared with placebo groups. Follow-up studies suggested existing structural heart disease, with ischemic and electrical instability present, to be the major risk factor for developing adverse reactions to flecainide.^177^ Later, the Survival with Oral d-Sotalol (SWORD) study raised concerns regarding the use of class III antiarrhythmic agents and demonstrated post-myocardial infarction patients at a higher risk of developing drug-induced arrhythmias.^178^ The same was shown for dronedarone, another class III antiarrhythmic, in the Antiarrhythmic Trial with Dronedarone in Moderate to Severe CHF Evaluating Morbidity Decrease (ANDROMEDA)^179^ and in the Permanent Atrial Fibrillation Outcome Study Using Dronedarone on Top of Standard Therapy (PALLAS).^180^

The cholesterol-lowering agent probucol, despite association with QT prolongation and arrhythmias,^82^ was discontinued from the US market due to lack of efficacy against coronary artery disease. Probucol affects hERG trafficking (Figure 4), and it was shown to cause electrophysiological abnormalities in neonatal rat ventricular cardiomyocytes.^112^

The antihypertension drug mibefradil is another example of a cardiovascular drug removed from the market, in 1998, because of safety concerns.^90^ Mibefradil blocks calcium channels and was shown to cause abnormal QT prolongation.^91^

### Other agents

A negative effect of antidiabetic drugs is their increased risk of heart failure, which has been confirmed in different studies.^181^ Considering such a risk, the European Medicines Agency (EMA) and FDA issued restrictions in 2010 for different drugs containing rosiglitazone, a thiazolidinedione-class antidiabetic drug. After a thorough examination of large clinical trials, the FDA removed those restrictions and eliminated the risk evaluation and mitigation strategy for rosiglitazone. Concerns regarding the safety of saxagliptin and alogliptin were also raised.

In 2007, the cough suppressant clobutinol was lifted from the market by its manufacturer due to risk of QT prolongation. Animal experiments suggested that clobutinol inhibits hERG channels and can induce torsade de pointes.^94^

### Conclusions

In this review, we have presented a comprehensive discussion of drug-protein mechanisms underlying clinical drug-induced cardiotoxicity. This significantly broadens other studies by covering adverse effects and cardiotoxic mechanisms of cardiovascular, anticancer, CNS, genitourinary system, gastrointestinal, antihistaminic, anti-inflammatory, and anti-infective agents beyond their direct interactions with cardiac electrophysiology. As a result, we illustrate that many of these drug classes share modes of action on cardiac function and on adverse cardiac events, with cardiotoxicity frequently resulting from the simultaneous interruption of key myocardial functions and viabilities (mitochondrial dysfunction, inhibition of major signaling pathways responsible for cardiomyocyte survival and maintenance, fibrosis, NO synthesis, calcium cycling, and cellular trafficking).

Understanding of the mechanisms of drug-induced toxicity and differences in cardiac safety profiles of therapies is important for the development of new compounds and of safety assays for preclinical testing. In the clinical scenario, the drug-induced mechanisms leading to adverse cardiac events are generally considered multifactorial in origin, and they often remain poorly understood. Therefore, a refined understanding of the multifaceted components of cardiotoxicity can lead to the development of new cardioprotective agents, with the potential of reducing the manifestation and damage of otherwise-effective but risky drugs. As an exemplar, statins have been proposed to lower anthracycline-induced harm via a reduction of ROS signaling and regulation of TOP2B.^182^ Similarly, the activation of the AMPK pathway by lapatinib may underlie its lower cardiotoxicity compared with other tyrosine kinase inhibitors. Other AMPK activators, such as metformin, have been shown to be protective for several heart conditions.^183^ Nevertheless, it is important to remember that cardiotoxicity highly depends on exposure levels (especially overdose) and the duration of treatment; thus, only some of the discussed adverse effects may be involved in clinical practice. For clinical applications such as oncology treatments, the risk-benefit ratios often overpower the consequences of potential cardiotoxic events.

This review focuses on mechanisms of drug-induced cardiac adverse effects, centering its scope on drug-protein interactions. Although certain mechanisms of action, such as tyrosine kinase inhibition or mitochondrial damage, could affect other cell types in the organism, in this review, we have centered our efforts on summarizing their known clinical manifestations on cardiac side effects. Drug-drug and drug-food interactions are also known to promote adverse cardiovascular effects, usually linked to competition among enzymes participating in drug metabolism or alterations in drug metabolism.^184^^,^^185^ In addition, recent advances in the annotation of RNA-RNA interactions suggest they might be of even higher importance in cardiac disease, such as heart failure and hypertrophy.^186^^,^^187^

All of these mechanisms of drug-induced cardiotoxicity constitute important and promising prospects for future research into drug discovery, safety pharmacology, and pharmacovigilance studies. Therefore, their integration as a priority into multi- and interdisciplinary approaches across academia, industry, and healthcare settings, for the complex characterization of human heart physiology,^188^^,^^189^ is expected to yield major advances for the analysis and prediction of adverse drug reactions and cardiotoxicity in humans, both in health and in disease.
